# Myxoid glioneuronal tumor, *PDGFRA* p.K385L‐mutant, arising in midbrain tectum with multifocal CSF dissemination

**DOI:** 10.1111/bpa.13008

**Published:** 2021-07-23

**Authors:** B. K. Kleinschmidt‐DeMasters, Jason Chiang, Andrew M. Donson, Thomas Borges, Ahmed Gilani

**Affiliations:** ^1^ Department of Pathology University of Colorado Anschutz Medical Campus Aurora CO USA; ^2^ Department of Neurology University of Colorado Anschutz Medical Campus Aurora CO USA; ^3^ Department of Neurosurgery University of Colorado Anschutz Medical Campus Aurora CO USA; ^4^ Department of Pathology St. Jude Children's Research Hospital Memphis TN USA; ^5^ Department of Pediatrics University of Colorado Anschutz Medical Campus Aurora CO USA; ^6^ Department of Radiology University of Colorado Anschutz Medical Campus Aurora CO USA

**Keywords:** brainstem, DNA methylation, glioneuronal, next generation sequencing, tumors

## Abstract

This myxoid glioneuronal tumor, *PDGFRA* p.K385L‐mutant, arose in the midbrain tectum rather than in the septum pellucidum, as in the previously‐reported cases.

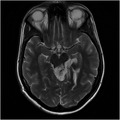

## CONFLICT OF INTEREST

The author(s) declared no potential conflicts of interest with respect to the research, authorship, and/or publication of this article.

## AUTHOR CONTRIBUTIONS

B.K. Kleinschmidt‐DeMasters: Acquisition, analysis, and interpretation of data for the work; drafting, revising and critical review. Jason Chiang: Acquisition, analysis, and interpretation of data for the work; revising and critical review. Andrew M. Donson: Acquisition, analysis, and interpretation of data for the work. Thomas Borges: Analyzing neuroimaging data. Ahmed Gilani: Acquisition, analysis, and interpretation of data for the work; drafting, revising, and critical review.

Myxoid glioneuronal tumor (MGNT) is a recently codified tumor with *PDGFRA* p.K385 mutation thus far epicentered/arising only in the septum pellucidum, septal nuclei, corpus callosum, and lateral ventricle. Histological features are similar to rosette‐forming glioneuronal tumor (RGNT) or dysembryoplastic neuroepithelial tumor (DNET) but, unlike those two entities, MGNT lack *PIK3CA/PIK3R1* alterations or *BRAF/FGFR1* mutations, respectively. We report the first genetically verified example of MGNT arising in the midbrain tectum, with additional unusual features of radiocontrast enhancement as well as multifocal infratentorial and spinal cord cerebrospinal fluid (CSF) dissemination at diagnosis.

**FIGURE 1:**
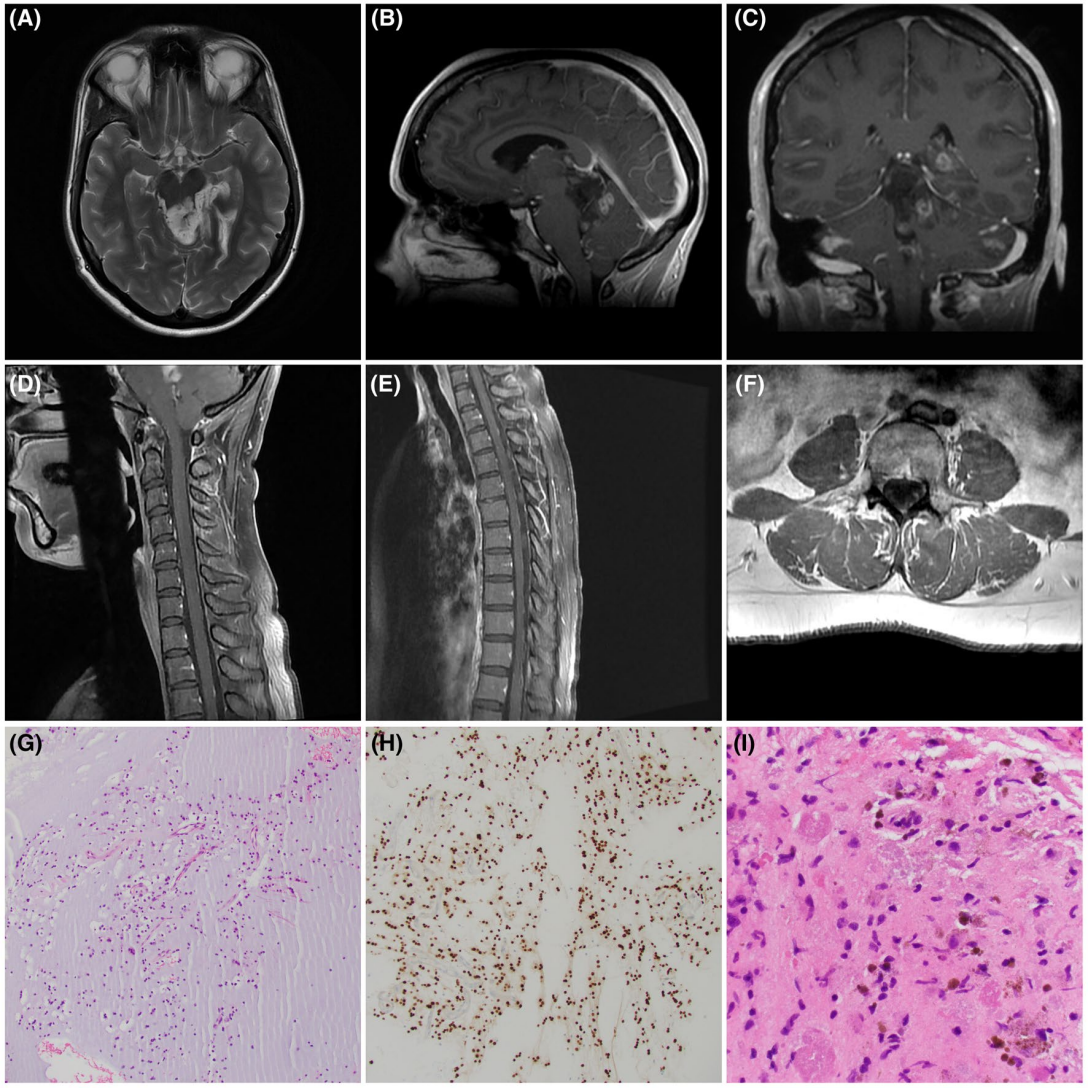
(A) Axial non‐contrast‐enhanced T2‐weighted MRI demonstrates the tectal plate origin (arrowhead) of the tumor. (B) Sagittal contrast‐enhanced T1‐weighted MRI proves the absence of corpus callosal or lateral ventricular involvement but highlights the enhancing component within the tectal tumor (arrowhead). (C) Coronal contrast‐enhanced T1‐weighted MRI shows the nodular posterior fossa spread (black arrowhead) as well as leptomeningeal enhancement within cerebellar folia (white arrowheads). (D) Sagittal contrast‐enhanced T1‐weighted MRI of cervical spine shows nodular enhancing masses over the dorsum of the cord (arrowheads). (E). Sagittal contrast‐enhanced T1‐weighted MRI of thoracic spine shows nodular enhancing masses over the dorsum of cord (arrowheads). (F) Axial post contrast T1‐weighted MRI shows nodular enhancement (arrowhead) involving the left L5 nerve roots. (G) Low power view of the small oligodendroglial‐like cells embedded in copious mucin (hematoxylin & eosin). (H) Tumor nuclei were diffusely immunoreactive for OLIG2 (IHC for OLIG2 with light hematoxylin counterstain). (I) Hemosiderin (arrowheads) and eosinophilic granular bodies (arrows) were focally identified (H&E)

A 41‐year‐old female developed headache and neck pain seven months prior to biopsy; progressive symptoms prompted her to seek medical attention. Neuroimaging demonstrated a complex exophytic lesion epicentered in midbrain tectum (Figure 1A, arrowhead), with enhancement (Figure 1B, arrowhead) and absence of involvement of corpus callosum or lateral ventricles (Figure 1B). Disseminated posteror fossa lesions included cerebellar nodular (arrowhead) and leptomeningeal enhancing spread (white arrowheads) (Figure 1C) as well as enhancing lesions over the dorsum of the cervical (Figure 1D, arrowheads) and thoracic cord (Figure 1E, arrowheads), and in L5 nerve root (Figure 1F, arrowhead). There was no tumor involvement of the septum verum (Figure S1). Histologic examination showed typical DNET‐like features, with uniform small round oligodendroglial‐like cells with delicate chromatin, embedded in copious mucin (Figure 1G). No floating neurons or rosettes were identified on H&E or synaptophysin immunohistochemical (IHC) staining; tumor cells manifested the characteristic scant GFAP+cytoplasm, strong diffuse nuclear OLIG2 (Figure 1H), and absent mitotic activity. Focal hemosiderin pigment (arrowheads) and eosinophilic granular bodies (arrows) were present (Figure 1I), a finding described in the minority of examples in the report by Chiang et al. (1). Mutational testing using a 300+ gene panel demonstrated *PDGFRA* p.K385L as well as a *NOTCH1* p.T1344M. The tumor was negative for clinically significant mutations or gene fusions involving *IDH1/2*, histone genes *H3F3A, H3F3B, HIST1H1C, HIST1H3B, HIST1H3C, BRAF, FGFR1, 2, 3, NF1, PIK3CA*, and *PIK3R1*. A complete list of genes tested in our 300+ gene fusion and mutation panels is included in the Supporting Information.

Similar to RGNT and DNET, originally reported in fourth ventricle and temporal lobe seizure specimens, respectively, but both subsequently identified elsewhere, we now report a MGNT arising in a mesencephalic location. Additional unusual features are the widespread CSF dissemination at presentation, including metastasis to the spinal cord and left L5 nerve roots.

As is always the case with newly diagnosed entities, there may be older similar examples in the literature reported prior to genetic testing becoming definitional of the entity. Specifically, we identified several literature examples of DNETs involving the tectum. These included a DNET of midbrain tectum reported in 2002 (2); a multinodular tumor of midbrain, bilateral mesial temporal lobes, diencephalon, and subependymal regions reported in 1999 (3); and a multifocal DNET in the left temporal lobe, third ventricle, and basal ganglia reported in 1994 (4). The case reported by Whittle et al. (3), however, manifested adjacent cortical dysplasia, a feature often seen near DNET, but not identified in the study by Chiang et al. (1). However, even if these older DNETs involving the tectum do represent MGNT, *PDGFRA* p.K385‐mutant, none had this degree of CSF dissemination.

In the series from St. Jude's Hospital, midbrain tectum involvement was not reported, although study design was restricted to archival cases of “septal DNET” (1). In that study, septum verum/septal nuclei was involved in all 15 patients from St. Jude's Hospital where imaging was available; the septum pellucidum itself was involved in only 7 of 15 St. Jude's cases (1). Literature review of 18 similar literature examples also showed septum verum/septal nuclei involved in all (1).

In addition to the anatomical origin in midbrain tectum in our case, without involvement of septum pellucidum, corpus callosum, lateral ventricle, or septal nuclei, the gadolinium enhancement is relatively uncommon. Specifically, “only 2 of 13 tumors evaluated with gadolinium contrast‐enhanced T1WI (T1WI + C) showed small nodular enhancement; otherwise, none enhanced” in the study by Chiang et al. (1).

The spinal cord dissemination in our case is unique. In the St. Jude's series, intraventricular dissemination was seen in 3 patients at presentation; “one later developed extensive leptomeningeal disease” (1). One additional case had documented leptomeningeal metastasis, but imaging was not available for review (1). Illustrations in the paper, however, show only lateral ventricular CSF spread; spinal metastases were not described. In the series of 8 patients reported by Lucas et al., 3 of 8 had disseminated intraventricular disease, 2 at initial presentation and 1 found later on serial imaging (5).

A final, but likely minor, unique feature of the current case is a second genetic alteration. There was evidence of a mutation in *NOTCH1*, resulting in an anticipated single amino acid substitution (p.T1344M). This variant has only been rarely reported in major cancer databases and has not been well‐described in CNS neoplasms. This variant does not occur within a functional domain of the protein product, and functional data is not readily available for this alteration. Therefore, the significance of this variant and its role in this patient's disease process is unknown.

Global DNA methylation has recently been shown to reduce inter‐observer variability and improve diagnostic precision in CNS tumor diagnosis (6). Given the histologic overlap between low‐grade tumors such as MGNT, DNET, and pilocytic astrocytoma, we performed DNA methylation profiling using standard methods (6) (Supporting Methods). Briefly, FFPE‐derived DNA was subjected to bisulfite treatment, followed by hybridization on Infinium MethylationEPIC BeadChip DNA methylation array. The output.idat files thus generated were uploaded to the German Cancer Research Center (DKFZ) web interface (classifier version: 11b2. https://www.molecularneuropathology.org/mnp/classifier/1, accessed: 06/15/2021). A methylation‐based classification and a chromosomal copy‐number plot were generated utilizing previously published algorithms (6). Similar to what is reported for other pediatric low‐grade neural tumors, including MGNT, there were minimal copy number variations (Figure S2). Methylation profiling using the Heidelberg CNS tumor classifier showed the highest similarity with DNET (calibrated score 0.46), as previously reported for MGNT (7). However, it fell short of the threshold (calibrated score ≥0.9) required to match this defined class. As proposed previously, failure to match a defined methylation class may indicate that the case in question represents a rare, possible location‐specific subtype (6).

Further assessment was conducted by one of the authors (JC). T‐SNE analysis of global DNA methylation data showed clustering of the tumor with tectal gliomas and not with septal DNET/MGNT (Figure S3). Tumor content was confirmed on histologic as well as molecular analysis, precluding the possibility of non‐tumor content affecting the DNA methylation data. It is noteworthy that *PDGFRA* altered DNET in septal and cortical locations showed distinct clusters as previously reported by Chiang et al. (1). The author (JC) then reviewed the histological features of all examples of tectal plate gliomas in his study and found none with DNET or MGNT‐like histology.

Although methylome profiles of CNS tumors largely depend upon tumor type and the molecular alteration present, leading to specific histopathologic entities clustering together even in anatomically different regions, RELA‐fusion positive ependymoma (8), and IDH‐mutant astrocytoma (9) being notable examples, other tumors form separate clusters based on tumor location (likely representing a different cell of origin) examples of which include anatomically unique pilocytic astrocytoma (10) and H3 K27M–mutant DIPG subgroups (9). In fact, recent papers have suggested that, for certain tumor groups, such as pediatric low‐grade gliomas, the epigenetic subgroups may be driven by nongenetic factors such as tumor location and nonneoplastic cell composition (10).

Until more MGNTs in non‐classical/non‐septum pellucidum sites are reported and studied by DNA methylation, it is impossible to know the full implications of our DNA methylation profiling results.

Broadly speaking, whether nosology of CNS tumors should follow the morphology, DNA methylation‐based clustering, genetic signature, or any combination of these factors remains a challenging question and should be addressed by the field in the coming years.

Clinical follow‐up is too short for meaningful interpretation in this case but no adjuvant therapy has thus far been administered.

## Supporting information


**FIGURE S1** Preoperative axial and coronal MR images showing the absence of tumor involvement in the septum verum. (A) T2 weighted axial, (B) T2‐FLAIR axial, (C) T1‐weighted axial, and (D) T2‐FLAIR coronal images

**FIGURE S2** Copy number variation profile obtained from methylation analysis. Gains/amplifications represent positive, losses negative deviations from the Sbaseline. 29 brain tumor‐relevant gene regions are highlighted for easier assessment

**FIGURE S3** Methylome profile of the case on the t‐SNE plot along with a reference cohort of CNS tumors showing clustering with tectal gliomas. AIDH, IDH‐mutant diffuse astrocytoma; CBPA, Cerebellar pilocytic astrocytoma; DNET, Dysembryoplastic Neuroepithelial Tumor; G34R, hemispheric glioblastoma with H3G34R mutation; GG, Ganglioglioma; HTPA, Hypothalamic pilocytic astrocytoma; K27M, H3K27M mutant glioma; OIDH, IDH‐mutant and 1p/19q‐codeleted oligodendroglioma; RGNT, rosette forming glioneuronal tumor; sDNET, MGNT/ septal DNET; SEGA, Subependymal giant cell astrocytoma; TG, tectal glioma

## Data Availability

Data sharing is not applicable to this article as no new data were created or analyzed in this study.
