# Supplementary material for: Myxoid glioneuronal tumor, PDGFRA p.K385L‐mutant, arising in midbrain tectum with multifocal CSF dissemination
Source: Brain Pathol. 2021 Jul 23;32(1):e13008. doi: 10.1111/bpa.13008 (PMC8713525; doi:10.1111/bpa.13008)

Supplementary Figure 1: Preoperative axial and coronal MR images showing the absence of tumor involvement in the septum verum. (A) T2 weighted axial, (B) T2-FLAIR axial, (C) T1-weighted axial, and (D) T2-FLAIR coronal images.


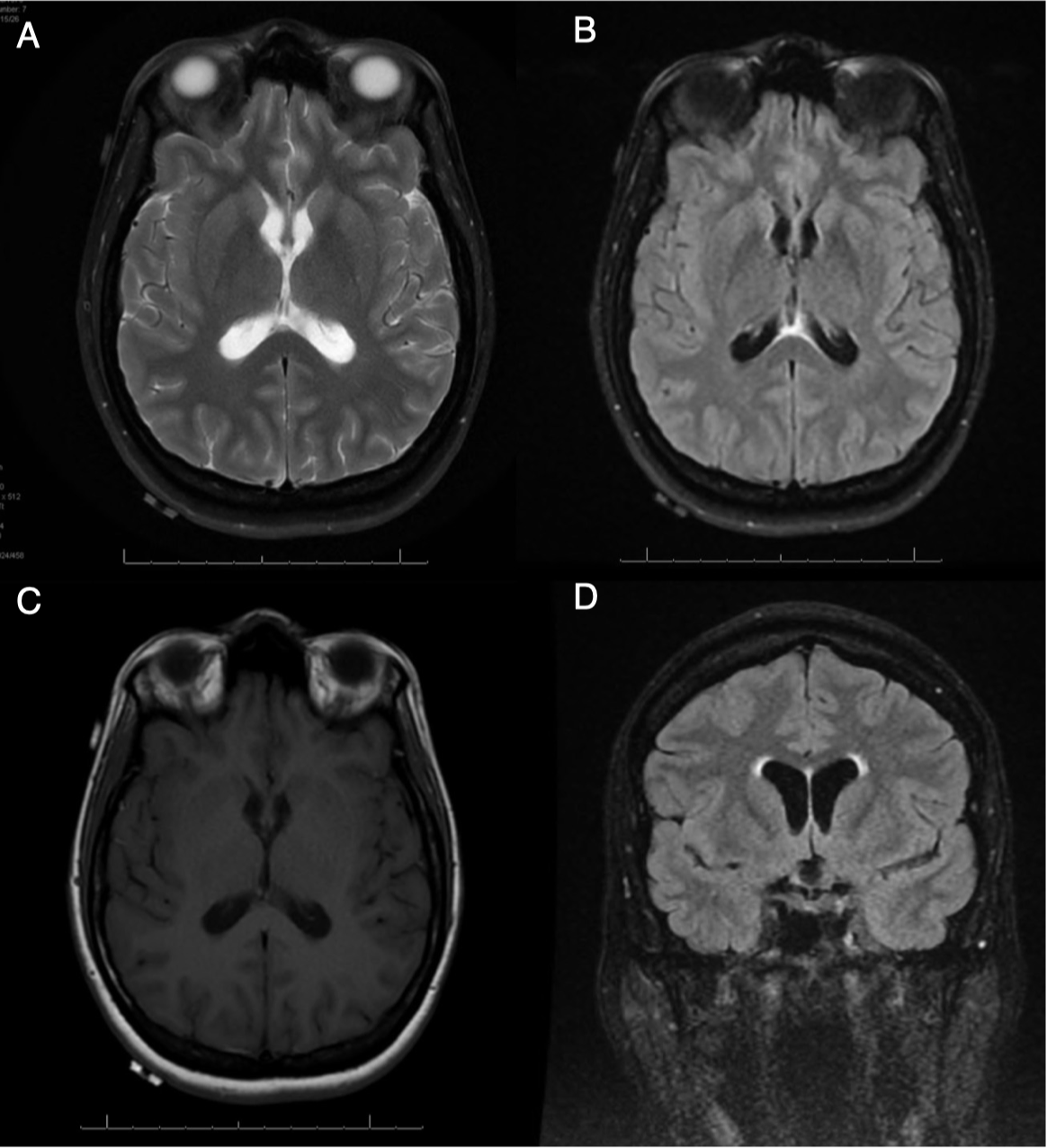

Supplement: Supplementary file 1 — FIGURE S1 Preoperative axial and coronal MR images showing the absence of tumor involvement in the septum verum. (A) T2 weighted axial, (B) T2‐FLAIR axial, (C) T1‐weighted axial, and (D) T2‐FLAIR coronal images [file BPA-32-e13008-s004.docx]
