# Supplementary material for: Myxoid glioneuronal tumor, PDGFRA p.K385L‐mutant, arising in midbrain tectum with multifocal CSF dissemination
Source: Brain Pathol. 2021 Jul 23;32(1):e13008. doi: 10.1111/bpa.13008 (PMC8713525; doi:10.1111/bpa.13008)

Supplementary Figure 2: Copy number variation profile obtained from methylation analysis. Gains/amplifications represent positive, losses negative deviations from the

baseline. 29 brain tumor-relevant gene regions are highlighted for easier assessment.


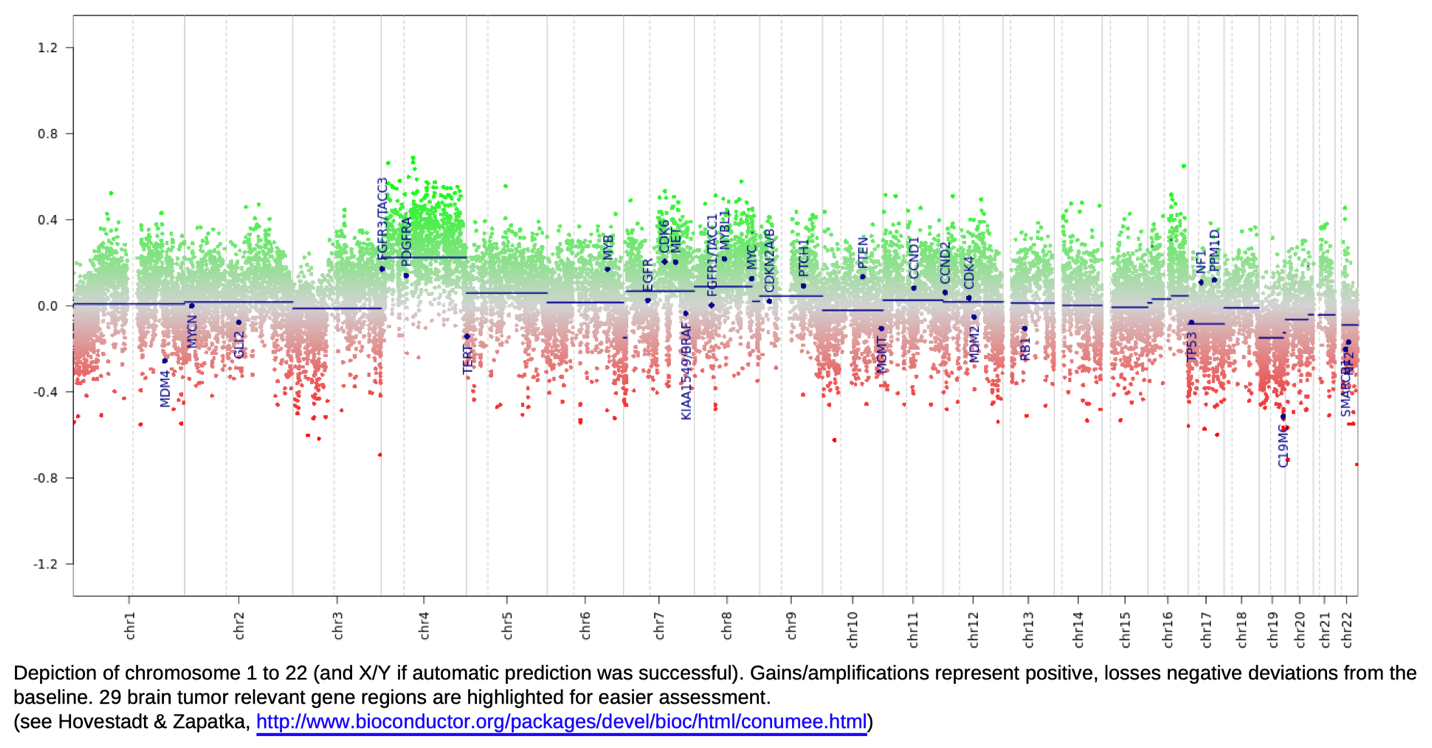

Supplement: Supplementary file 2 — FIGURE S2 Copy number variation profile obtained from methylation analysis. Gains/amplifications represent positive, losses negative deviations from the Sbaseline. 29 brain tumor‐relevant gene regions are highlighted for easier assessment [file BPA-32-e13008-s001.docx]
