# Supplementary material for: Myxoid glioneuronal tumor, PDGFRA p.K385L‐mutant, arising in midbrain tectum with multifocal CSF dissemination
Source: Brain Pathol. 2021 Jul 23;32(1):e13008. doi: 10.1111/bpa.13008 (PMC8713525; doi:10.1111/bpa.13008)

Supplementary Figure 3: Methylome profile of the case on the t-SNE plot along with a reference cohort of CNS tumors showing clustering with tectal gliomas. Abbreviations: AIDH: IDH-mutant diffuse astrocytoma, CBPA: Cerebellar pilocytic astrocytoma, DNET: Dysembryoplastic Neuroepithelial Tumor, G34R: hemispheric glioblastoma with H3G34R mutation, GG: Ganglioglioma, HTPA: Hypothalamic pilocytic astrocytoma, K27M: H3K27M mutant glioma, OIDH: IDH-mutant and 1p/19q-codeleted oligodendroglioma, RGNT: rosette forming glioneuronal tumor, sDNET: MGNT/ septal DNET, SEGA: Subependymal giant cell astrocytoma, TG: tectal glioma.


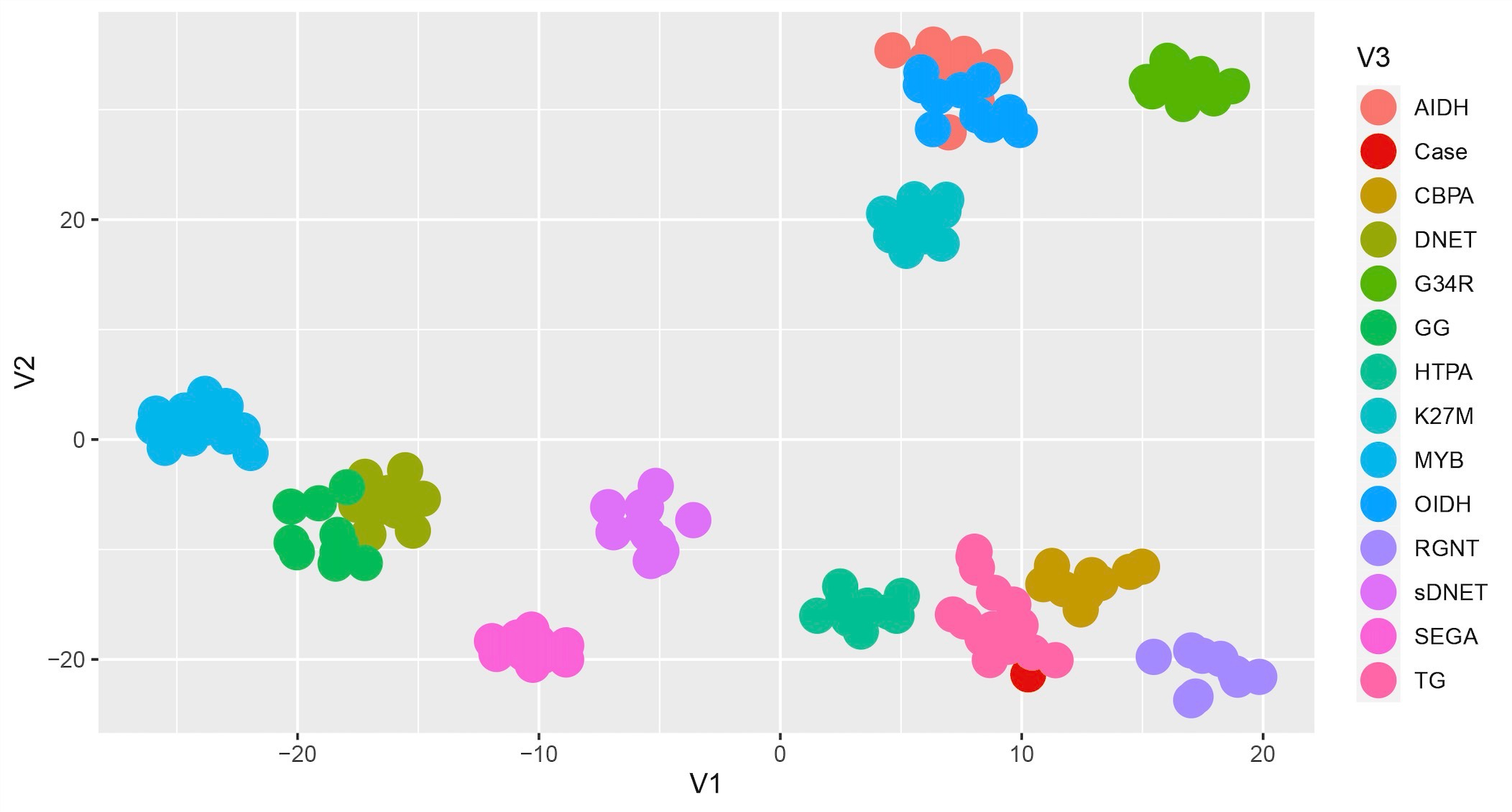

Supplement: Supplementary file 3 — FIGURE S3 Methylome profile of the case on the t‐SNE plot along with a reference cohort of CNS tumors showing clustering with tectal gliomas. AIDH, IDH‐mutant diffuse astrocytoma; CBPA, Cerebellar pilocytic astrocytoma; DNET, Dysembryoplastic Neuroepithelial Tumor; G34R, hemispheric glioblastoma with H3G34R mutation; GG, Ganglioglioma; HTPA, Hypothalamic pilocytic astrocytoma; K27M, H3K27M mutant glioma; OIDH, IDH‐mutant and 1p/19q‐codeleted oligodendroglioma; RGNT, rosette forming glioneuronal tumor; sDNET, MGNT/ septal DNET; SEGA, Subependymal giant cell astrocytoma; TG, tectal glioma [file BPA-32-e13008-s002.docx]
