# Supplementary material for: Myxoid glioneuronal tumor, PDGFRA p.K385L‐mutant, arising in midbrain tectum with multifocal CSF dissemination
Source: Brain Pathol. 2021 Jul 23;32(1):e13008. doi: 10.1111/bpa.13008 (PMC8713525; doi:10.1111/bpa.13008)
Supplement: Supplementary file 4 [file BPA-32-e13008-s003.docx]

Supplementary methods

Next-generation sequencing:

Fusion and mutation testing was performed in a clinical laboratory improvement amendments (CLIA)-certified laboratory environment. Nucleic acids were extracted from formalin- fixed, paraffin-embedded tissue blocks processed for routine histology. Extracted nucleic acids were subjected to a hybrid capture step targeting the entire coding regions of over 300 genes for DNA and over 500 genes for RNA (see lists of genes below).  Next-generation sequencing was performed on the Illumina Novaseq 6000.  Data was analyzed using an in-house developed pipeline that filters the data for quality and assigns variant calls.  Interpretation was then performed within a specialized software environment that allows for the integration of variant databases, in silico modeling, and published literature to assign a clinical significance for each variant in the appropriate tumor context.  The DNA panel is able to detect single nucleotide variants and small insertion/deletion variants.  The RNA panel is able to detect fusion transcripts, including novel transcripts as long as one partner is captured on the panel.  These panels have been specifically designed to target pediatric neoplasms.

Methylation Array

Genomic DNA was isolated from formalin fixed paraffin embedded tissue blocks that were processed for routine histologic diagnosis, using Qiagen DNA FFPE Tissue Kit. DNA methylation was assessed using the Infinium® MethylationEPIC BeadChip (Illumina). 250 ng of DNA was submitted for bisulfite conversion using Zymo EZ DNA Methylation kits (Zymo Research Corp). Following the manufacturer’s protocol, bsDNA was, amplified, fragmented and hybridized to BeadChips. The chips were stained and then read with an iScan (Illumina), generating .idat files to be used in subsequent statistical analyses. IDAT files were batch normalized and background corrected using default settings of the R package ChAMP. Resulting IDAT files were uploaded to MolecularNeuropathology.org (https:// [www.molecularneuropathology.org/mnp](http://www.molecularneuropathology.org/mnp)). Copy number analysis data were generated from unnormalized signal intensities using ChAMP module with default control samples.

DNA panel: This panel includes 301 genes with clinical significance for the diagnosis, prognosis, and therapeutic decision making for solid, hematopoietic and central nervous system tumors.

Gene content: *ABL2, ACVR1, ACVRL1, AKT1, AKT2, AKT3, ALK, AMER1, ANKRD26, APC, ARAF, ARID1A, ARID1B, ARID2, ASXL1, ASXL2, ATM, ATR, ATRX, AURKA, AURKB, AXIN1, B2M, BCL11B, BCL2, BCL6, BCOR, BCORL1, BRAF, BRCA1, BRCA2, BRD4, BRIP1, CALR, CARD11, CBL, CBLB, CCBE1, CCM2, CCND1, CCND2, CCND3, CCNE1, CCR4, CCR7, CD274, CD58, CD79A, CD79B, CDK12, CDK4, CDK6, CDK8, CDKN1B, CDKN1C, CDKN2A, CDKN2B, CDKN2C, CEBPA, CHD7, CHEK1, CHEK2, CIC, CIITA, CREBBP, CRLF2, CSF1R, CSF3R, CTNNB1, CUX1, CXCR4, DAXX, DDR2, DDX3X, DDX41, DICER1, DIS3L2, DLG2, DNM2, DNMT3A, EBF1, EED, EGFR, ELMO2, ENG, EP300, EPHA7, EPHB4, ERBB2, ERBB3, ERBB4, ERG, ESR1, ETV6, EZH2, FANCA, FANCC, FAS, FASLG, FBXW7, FGFR1, FGFR2, FGFR3, FGFR4, FLCN, FLT1, FLT3, FLT4, FOXC2, FOXO1, FOXP1, FUBP1, GATA1, GATA2, GATA3, GJC2, GLI1, GLI2, GLMN, GNA11, GNA13, GNA14, GNAQ, GNAS, GPC3, H3F3A, H3F3B, HIST1H1C, HIST1H3B, HIST1H3C, HRAS, ID3, IDH1, IDH2, IGF1R, IKZF1, IKZF3, IL7R, INO80, IRS1, JAK1, JAK2, JAK3, JUN, JUNB, KDM4C, KDM5A, KDM5C, KDM6A, KDR, KIF11, KIT, KLF2, KMT2A, KMT2C, KMT2D, KRAS, KRIT1, LZTR1, MAP2K1, MAP2K2, MAP2K4, MAP3K1, MAP3K3, MAPK1, MDM2, MDM4, MECOM, MEF2B, MEN1, MET, MLH1, MPL, MSH2, MSH6, MTOR, MYB, MYBL1, MYC, MYCN, MYD88, MYOD1, NBN, NCOR2, NF1, NF2, NOP10, NOTCH1, NOTCH2, NPM1, NRAS, NT5C2, NTRK1, NTRK2, NTRK3, PAX5, PDCD1, PDCD10, PDGFRA, PDGFRB, PHF6, PHOX2B, PIGA, PIK3CA, PIK3CB, PIK3CG, PIK3R1, PIK3R2, PIM1, PML, PMS2, POLE, PPM1D, PRDM1, PRPS1, PSMB5, PTCH1, PTEN, PTPN1, PTPN11, PTPN14, PTPRD, RAD21, RAD51, RAD51C, RAF1, RASA1, RB1, REL, RELN, RET, RHOA, RICTOR, RNF125, RNF135, ROS1, RPTOR, RRAS, RUNX1, SAMD9, SAMD9L, SDHA, SDHB, SDHC, SDHD, SETBP1, SETD2, SF3B1, SGK1, SH2B3, SMAD2, SMAD3, SMAD4, SMARCA4, SMARCB1, SMC1A, SMC3, SMO, SOCS1, SOX18, SRC, SRSF2, STAG2, STAMBP, STAT3, STAT5B, STAT6, STK11, SUFU, SUZ12, TCF3, TEK, TENT5c (FAM46C), TERC, TERT, TET2, TET3, TGFBR2, TINF2, TLX1, TNFAIP3, TNFRSF10A, TNFRSF14, TP53, TP63, TSC1, TSC2, U2AF1, UBE2T, USP7, VEGFC, VHL, WHSC1, WT1, XPO1, ZEB1, ZMYM3, ZRSR2, hist1H1C,ABL1,ACVR1*

RNA panel: This panel is able to detect a fusion event as long as one of the genes involved is present on the panel.

Gene content: *ABI1, ABL1, ABL2, ACACA, ACE, ACER1, ACKR3, ACSL6, ACTB, ADD3, AFF1, AFF3, AFF4, AGR3, AHI1, AHRR, ALK, ANKRD28, AR, ARHGAP20, ARHGAP26, ARNT, ASPSCR1, ASTN2, ATF1, ATIC, ATP1B4, AUTS2, AXL, BACH2, BAG4, BAIAP2L1, BAZ2A, BCAS3, BCAS4, BCL10, BCL11A, BCL11B, BCL2, BCL2L1, BCL3, BCL6, BCL9, BCOR, BCR, BDNF, BICC1, BIRC3, BIRC6, BLNK, BRAF, BRD1, BRD3, BRD4, BRWD3, BTBD18, BTG1, C11orf1, C11orf95, C2CD2L, CAMTA1, CAPRIN1, CARS, CASC5, CASP7, CBFA2T3, CBFB, CBL, CCAR2, CCDC28A, CCDC6, CCDC88C, CCNB1IP1, CCNB3, CCND1, CCND2, CCND3, CD74, CDH11, CDK5RAP2, CDK6, CDX1, CDX2, CEBPA, CEBPB, CEBPD, CEBPE, CEP170B, CEP85L, CHD6, CHIC2, CHMP2B, CHST11, CIC, CIITA, CITED2, CLP1, CLTC, CLTCL1, CMKLR1, CNBP, CNOT2, CNTRL, COG5, COL1A1, COL1A2, COL6A3, COX6C, CPSF6, CRADD, CREB1, CREB3L1, CREB3L2, CREBBP, CRLF1, CRLF2, CRTC1, CSF1, CSF1R, CTDSP2, CTNNB1, CUX1, DAB2IP, DACH1, DACH2, DDIT3, DDX10, DDX20, DEK, DGKH, DMRT1, DNAJB1, DPM1, DUSP22, EBF1, EEFSEC, EGFR, EGR1, EGR2, EGR3, EGR4, EIF4A2, ELF4, ELK4, ELL, ELN, EML1, EML4, EP300, EP400, EPC1, EPOR, EPS15, ERBB2, ERBB3, ERC1, ERCC1, ERG, ERLIN2, ESR1, ETS1, ETV1, ETV4, ETV5, ETV6, EVL, EWSR1, EZR, FAM19A2, FCGR2B, FCRL4, FEN1, FEV, FGF8, FGFR1, FGFR1OP, FGFR1OP2, FGFR2, FGFR3, FGFR4, FGR, FHIT, FIP1L1, FLI1, FLNA, FLT3, FLT3LG, FNBP1, FOS, FOSB, FOSL1, FOXO1, FOXO4, FOXP1, FRK, FRYL, FUS, GAS7, GATA1, GIT2, GLI1, GLIS2, GOSR1, GOT1, GPR128, GPR34, GRHPR, GRID1, GRM1, GTF2I, H2AFX, HAS2, HEY1, HHEX, HIP1, HIPK1, HIST1H4I, HLF, HMGA2, HNF1A, HOXA10, HOXA11, HOXA13, HOXA9, HOXC11, HOXC13, HOXD11, HOXD13, HRAS, HSP90AA1, ID4, IKZF1, IKZF2, IKZF3, IL2, IL21R, IL2RB, IL3, INPP5D, INSR, IQCG, IRF2BP2, IRF4, IRS4, ITK, JAK1, JAK2, JAZF1, KANK1, KAT6A, KAT6B, KDM5A, KIAA1524, KIF5B, KMT2A, KMT2B, KMT2C, KMT2D, KPNB1, KRAS, KSR1, LASP1, LCK, LCP1, LGR5, LHFP, LHX2, LHX4, LMBRD1, LMO1, LMO2, LNP1, LPP, LPXN, LRMP, LRRC37B, LTBP1, LYL1, MACROD1, MAF, MAFB, MALT1, MAML2, MAN2B1, MAPRE1, MBNL1, MBTD1, MDS2, MEAF6, MECOM, MEF2D, MET, MGEA5, MKL1, MKL2, MLF1, MLLT1, MLLT10, MLLT11, MLLT3, MLLT4, MLLT6, MN1, MNX1, MSI2, MSN, MTCP1, MUC1, MUTYH, MYB, MYBL1, MYC, MYH11, MYH9, MYO18A, MYO1F, NAB2, NAPA, NBR1, NCOA1, NCOA2, NCOA3, NCOR1, NDE1, NF1, NFATC2, NFIB, NGF, NGFR, NIN, NIPBL, NKX2-1, NKX2-5, NONO, NOTCH1, NOTCH2, NPM1, NR4A3, NR6A1, NRAS, NRG1, NSD1, NT5C2, NTF3, NTF4, NTRK1, NTRK2, NTRK3, NUMA1, NUMBL, NUP107, NUP214, NUP98, NUTM1, NUTM2A, NUTM2B, OFD1, OLIG2, OLR1, OMD, P2RY8, PAPPA, PATZ1, PAX3, PAX5, PAX7, PAX8, PBX1, PCM1, PDE4DIP, PDGFB, PDGFRA, PDGFRB, PER1, PGR, PHF1, PHF23, PICALM, PIK3CA, PIM1, PKN1, PLAG1, PML, POM121, POU2AF1, POU5F1, PPAP2B, PPARG, PPARGC1A, PPFIBP1, PPP2R1B, PRCC, PRDM16, PRKACA, PRKAR1A, PRKCA, PRKCB, PRKG2, PRRX2, PSIP1, PSMD2, PTK2B, PTPRR, RABEP1, RAD51B, RAF1, RANBP2, RAP1GDS1, RARA, RBM15, RBM6, RCOR1, RCSD1, RELA, RET, RHOH, RNF213, ROS1, RPL22, RPN1, RREB1, RRM1, RSPO2, RSPO3, RTEL1, RUNX1, RUNX1T1, SARNP, SEC31A, SEPTIN2, SEPTIN5, SEPTIN6, SEPTIN9, SERPINE1, SERPINF1, SET, SETBP1, SFPQ, SH3D19, SH3GL1, SIK3, SLC34A2, SLC45A3, SLCO1B3, SMAP1, SMARCA5, SMARCB1, SNHG5, SORBS2, SORT1, SP3, SPECC1, SPTBN1, SQSTM1, SRF, SRSF3, SS18, SS18L1, SSBP2, SSX1, SSX2, SSX4, ST6GAL1, STAT5B, STAT6, STIL, STRN, SUFU, SUGP2, SUZ12, SYK, TACC1, TACC2, TACC3, TAF15, TAL1, TAL2, TAOK1, TBX15, TCF12, TCF3, TCL1A, TCTA, TEAD1, TEAD2, TEAD3, TEAD4, TEC, TENM1, TERT, TET1, TFE3, TFEB, TFG, TFPT, TFRC, TGFBR3, THADA, THRAP3, TIRAP, TLX1, TLX3, TMPRSS2, TNFRSF17, TOP1, TOP2B, TP53BP1, TP63, TP73, TPM3, TPM4, TRA, TRB, TRD, TRHDE, TRIM24, TRIP11, TRPS1, TSLP, TTYH1, TYK2, USP16, USP42, USP6, VGLL2, VGLL3, WASF2, WDR18, WDR70, WHSC1, WHSC1L1, WSB1, WT1, WWTR1, XIAP, YAP1, YTHDF2, YWHAE, ZBTB16, ZC3H7A, ZC3H7B, ZFP64, ZFPM2, ZFYVE19, ZMIZ1, ZMYM2, ZMYND11, ZNF207, ZNF384, ZNF444, ZNF521, ZNF585B, ZNF687*
